# Systemic Propagation of a Fluorescent Infectious Clone of a Polerovirus Following Inoculation by Agrobacteria and Aphids

**DOI:** 10.3390/v9070166

**Published:** 2017-06-29

**Authors:** Sylvaine Boissinot, Elodie Pichon, Céline Sorin, Céline Piccini, Danièle Scheidecker, Véronique Ziegler-Graff, Véronique Brault

**Affiliations:** 1 Université de Strasbourg, Institut National de la Recherche Agronomique, SVQV UMR-A 1131, 68000 Colmar, France; sylvaine.boissinot@inra.fr (S.B.); elodie.pichon@inra.fr (E.P.); 2 UMR 385 BGPI, Institut National de la Recherche Agronomique—Centre de Coopération Internationale en Recherche Agronomique pour le Développement, SupAgro, CIRAD TA-A54/K, Campus International de Baillarguet, 34398 Montpellier, France; 3 Institut de Biologie Moléculaire des Plantes, Centre National de la Recherche Scientifique, UPR 2357, Université de Strasbourg, 12 rue du Général Zimmer, 67084 Strasbourg, France; celine.sorin@ips2.universite-paris-saclay.fr (C.S.); celine.piccini@hotmail.com (C.P.); daniele.scheidecker@ibmp-cnrs.unistra.fr (D.S.); veronique.ziegler-graff@ibmp-cnrs.unistra.fr (V.Z.-G.); 4 Institute of Plant Science Paris Saclay (IPS2), CNRS, INRA, University Paris Diderot, University of Paris-Saclay, 91405 Orsay, France

**Keywords:** polerovirus, fluorescent clone, systemic infection, aphid transmission

## Abstract

A fluorescent viral clone of the polerovirus Turnip yellows virus (TuYV) was engineered by introducing the Enhanced Green Fluorescent Protein (EGFP) sequence into the non-structural domain sequence of the readthrough protein, a minor capsid protein. The resulting recombinant virus, referred to as TuYV-RT_GFP_, was infectious in several plant species when delivered by agroinoculation and invaded efficiently non-inoculated leaves. As expected for poleroviruses, which infect only phloem cells, the fluorescence emitted by TuYV-RT_GFP_ was restricted to the vasculature of infected plants. In addition, TuYV-RT_GFP_ was aphid transmissible and enabled the observation of the initial sites of infection in the phloem after aphid probing in epidermal cells. The aphid-transmitted virus moved efficiently to leaves distant from the inoculation sites and importantly retained the EGFP sequence in the viral genome. This work reports on the first engineered member in the *Luteoviridae* family that can be visualized by fluorescence emission in systemic leaves of different plant species after agroinoculation or aphid transmission.

## 1. Introduction

Turnip yellows virus (TuYV, formerly BWYV-FL1) is a member of the *Polerovirus* genus in the *Luteoviridae* family. Its single-stranded positive sense RNA genome of approximately 6 kb is encapsidated into isometric particles of 25 nm in diameter. TuYV has a wide host range among herbaceous plants and infects important crops such as oilseed rape [1]. The genome consists of seven interlocked and overlapping open reading frames (ORFs), which are expressed from the genomic and subgenomic RNAs by non-canonical translation mechanisms [2]. Members of the *Luteoviridae* are strictly restricted to the three cell types constituting the phloem; the nucleated phloem parenchyma cells and companion cells, where the virus replicates, and the sieve elements, which convey the virus to sites distant from the inoculation point [3,4,5,6]. TuYV is obligatorily transmitted by aphids in a circulative and non-propagative mode [7]. The virus, acquired by the aphid while ingesting phloem sap from an infected plant, is transported successively through the intestinal epithelium and the accessory salivary gland cells before being released into a plant along with the saliva [8].

Using site-directed mutagenesis on a full-length TuYV infectious clone, specific roles have been attributed to the different virus-encoded proteins; P0 is the viral silencing suppressor that counteracts the plant defense pathway by degrading ARGONAUTE1 a key enzyme of the RNA silencing machinery [9,10,11,12,13]. P1 and P2 contain domains corresponding to a serine protease, the viral genome-linked protein (VPg), a helicase, and the polymerase [14,15]. The proteins encoded by the ORFs located at the 3′ end of the genome are expressed from a subgenomic RNA. ORF3 encodes the major coat protein (CP) and ORF5 the readthrough domain (RTD), which is expressed by a readthrough mechanism of the CP stop codon. This process results in the synthesis of a fusion protein, referred to as the readthrough protein (RT), containing the CP in its N-terminal part and the RTD in its C-terminus. A C-terminally truncated form of the RT, named RT*, is present as a minor component in the virus particle [16,17,18]. Poleroviruses CP and RT are involved in virus movement, and the RT* is strictly required for aphid transmission [5,17,19,20,21,22,23,24,25]. ORF4, embedded in ORF3, encodes a host-specific movement protein [26,27,28,29]. Very recently, a short ORF expressed from a non-canonical initiation codon and referred to as ORF3a was identified by in silico analyses. The encoded protein of about 5 kDa was shown to be essential for TuYV long-distance movement [2].

Up to now, TuYV localization in infected plants was only achieved by observing whole virions by transmission electron microscopy or by detecting the major structural protein by in situ immunolocalization using specific antibodies [3,4,5]. Although these techniques are informative and contributed to deciphering the role of the RT protein in TuYV movement, they are laborious and time consuming. Moreover, these destructive methods limit the monitoring of the virus progression kinetics after inoculation. In order to gain a better understanding of polerovirus movement in plants, we engineered an Enhanced Green Fluorescent Protein EGFP-tagged full-length infectious clone of TuYV. Only two Green Fluorescent Protein (GFP)-labelled poleroviruses have been reported so far, but none of them were able to stably infect systemically the inoculated plants [30,31]. The difficulty in obtaining a fluorescently-tagged polerovirus resides in the introduction of extra sequences into the dense genome containing several overlapping ORFs, without altering virus infectivity. Using former and recent data on the viral sequences required for the readthrough mechanism of the CP stop codon, for the encapsidation of the RT* into the viral particles and for virus efficient movement in plants, we designed a TuYV fluorescent clone by replacing the C-terminal part of the RT protein with the EGFP sequence. When delivered by agroinoculation or by aphids, TuYV-RT_GFP_ was able to invade the non-inoculated leaves of host species belonging to three plant families. As expected for a phloem-limited virus, the fluorescence emitted by TuYV-RT_GFP_ was restricted to phloem cells in those hosts. Interestingly, the initial phloem infection sites following aphid probing in epidermal cells were also observed. This represents the first report of a tagged virus in the *Luteoviridae* family that is able to develop a systemic infection after delivery by aphids or agroinfiltration.

## 2. Materials and Methods

### 2.1. TuYV Constructs

TuYV-RT_GFP_ was constructed by replacing the *Nco*I-*Sal*I fragment corresponding to the 3’ end of the TuYV sequence (nt 4822-downstream of the viral sequence) with a modified fragment containing the EGFP sequence [32] flanked by viral sequences. This fragment was built by regular restriction-ligation cloning of three PCR fragments into pBluescript KS plasmid (Stratagene). A first PCR fragment corresponding to the viral sequence 4822–4946 flanked by an *Nco*I site and a *Bam*HI site was introduced into pKS digested with *Nco*I and *BamH*I enzymes. A second PCR fragment corresponding to the viral sequence 5469–5641 flanked by *EcoR*I sites and *Sal*I sites, respectively, was inserted in the corresponding sites of the former vector. Finally, the EGFP sequence flanked by *BamH*I and *EcoR*I sites was introduced between the two former fragments in the recombinant pKS plasmid. The resulting *Nco*I-*Sal*I fragment was excised and used to replace the corresponding fragment in the pBW. A- vector [33], generating pTuYV-RT_GFP_. The primers used in the cloning are indicated in Table S1. In order to reconstitute the agroinfection binary vector pbinTuYV-RT_GFP_ containing full-length TuYV cDNA with the EGFP sequence, the *Spe*I/*Sal*I fragment of pTuYV-RT_GFP_ was excised and used to replace the corresponding sequence of pbinTuYV-WT. All constructs were verified by sequencing.

### 2.2. Protoplasts and Plant Infection

*Chenopodium quinoa* protoplasts were prepared and inoculated as described by Ziegler-Graff et al. [29]. 30 µg of plasmid pTuYV-WT (former pBW.A-, [33]) or pTuYV-RT_GFP_ were electroporated to 250,000 protoplasts using a pulse of 200 V. The protoplasts were harvested 48 h post-inoculation, and the RNA and proteins contents were analyzed.

The plants were either inoculated by agrobacteria or by viruliferous aphids. For agroinoculation, pbin plasmids containing the viral sequences were introduced into *Agrobacterium tumefaciens* C58C1 [34], and a cell culture was grown to an OD_600_ of 0.5 before being agroinfiltrated into three to five week old *Arabidopsis thaliana*, *Montia perfoliata*, or *Nicotiana benthamiana* [35]. For virus inoculation by aphids, virus suspensions were purified from *M. perfoliata* agroinoculated leaves, following the procedure described by van den Heuvel et al. [36]. Purified virus particles were observed by immunosorbent electron microscopy as described previously [37]. After a 24 h acquisition period on an artificial medium containing 100 ng/µL of virus in MP148 [22], 10 *Myzus persicae* (Sulzer) were transferred onto *M. perfoliata* or *A. thaliana* for virus inoculation. Alternately, 30 *M. persicae* ssp. *nicotianae* were transferred onto *N. benthamiana*. Four days after inoculation, the aphids were eliminated with Pirimor (Guyancourt, France) insecticide (0.5 mg/mL). When the aphid-inoculated leaves were used for microscopic observations, the aphids were fed on purified virus at a concentration set up to 300 ng/µL before being enclosed in clip-cages (10 aphids per clip-cage) deposited on *M. perfoliata*. The aphids were then removed with a brush before observations.

### 2.3. Detection of Viral RNA and Capsid Proteins

Total RNAs were extracted from protoplasts as described by Veidt et al. [38]. Viral RNA was detected by Northern blot using a ^32^P-dCTP labelled PCR probe complementary to a domain common to TuYV-WT and TuYV-RT_GFP_ (nt 4411–4827). RNA loading was controlled by staining the membrane using methylene blue. RNA quantification on Northern blots was performed using ImageGauge software (Fujifilm, Saint-Quentin-en-Yvelines, France) and the ImageJ processing program (https://imagej.nih.gov, Rockville Pike, MD, USA). The presence of the EGFP sequence in the viral progeny of the infected plants was analysed following the extraction of total RNA from the plant tissue using a commercial RNA purification kit (NucleospinRNA plant, Macherey Nagel). RNA was then subjected to reverse transcription (M-MLV reverse transcriptase; Promega), with the reverse primer (RP) complementary to the 3’-untranslated region of TuYV (Table S1). PCR-amplified fragments were obtained using the primer RP and the forward primer FP (Table S1) in the ORF5 sequence of TuYV viruses.

Viral proteins were detected in protoplasts or in agroinfiltrated *N. benthamiana* leaves by western blot using a polyclonal antisera raised against the TuYV-CP protein [22] and in non-inoculated leaves by double-antibody sandwich enzyme-linked immunosorbent assay (DAS-ELISA) with a TuYV-specific polyclonal antiserum (Loewe) [39]. Alternately, a homemade antiserum raised against the GFP protein was used. Briefly, protoplasts were directly disrupted using Laemmli buffer [40], and plant proteins were first ground with glass beads in liquid nitrogen before being denatured using Laemmli buffer and centrifuged for 5 min at 10,000 rpm. Proteins separated on a 10% or 11% sodium dodecyl sulfate polyacrylamide gel electrophoresis (SDS-PAGE) were transferred on a polyvinylidene difluoride (PVDF) Immobilon-P membrane (Merck-Millipore, Molsheim, France) and further incubated with primary antibodies (mentioned above). After the addition of a secondary antibody (goat-anti rabbit-HRP conjugate, Invitrogen, Camrillo, CA, USA) and HRP substrate (Lumi-LightPLUS kit, Roche, Mannheim, Germany) the protein/antibody complex was detected with a cooled high sensitive camera (Gbox, Syngene, Cambridge, United Kingdom) or by autoradiography. For loading controls, the nitrocellulose membranes were stained with Ponceau Red.

### 2.4. Epifluorescence Microscopy

Hand-cut cross and longitudinal sections of stems and petioles were mounted in water. To observe *A. thaliana* veins, the upper side of the leaf was stuck on a tape and the epidermal and mesophyll cells of the other face were removed with a second tape, thus releasing the vascular tissue. The vein network was then collected with tweezers and mounted in water. GFP fluorescence was observed using an Axio Imager M2 microscope (Zeiss, Marly le Roi, France) equipped with a Hamamatsu digital camera. Observations were performed on non-inoculated leaves of *N. benthamiana*, *A. thaliana*, and *M. perfoliata* two to three weeks post-infection, on inoculated leaves of *N. benthamiana* after agroinfiltration, or on *M. perfoliata* after virus inoculation by aphids. Some of the observations are optical sections obtained using structured illumination (ApoTome, Zeiss). For macroscopic observations, the leaves were observed on whole plants with an epifluorescence macroscope (Axiozoom.V16, Zeiss) equipped with a colour camera (Axiocam 506 color, Zeiss).

## 3. Results

### 3.1. Infectivity of TuYV-RT_GFP_ in Protoplasts and Inoculated Leaves

**Figure 1:**
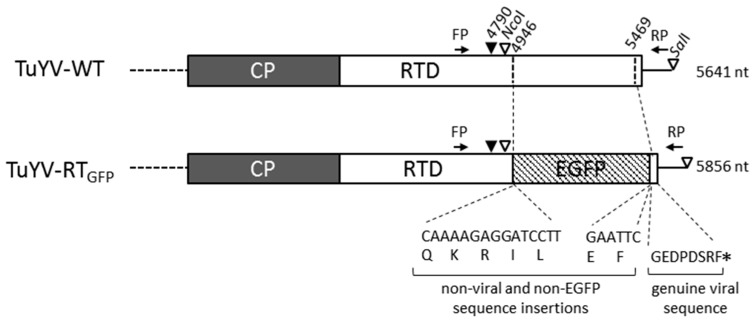
Schematic representation of the coat protein (CP) and the readthrough domain (RTD)-encoded open reading frame (ORF) at the 3′ of the wild-type polerovirus *Turnip yellows virus* (TuYV) TuYV-WT and the recombinant TuYV-RT_GFP_. The position of nucleotides on the TuYV genome is indicated. Extra non-viral and non-EGFP (enhanced green fluorescent protein) nucleotides introduced in the TuYV-RT_GFP_ genome and the corresponding amino acids are shown together with the viral-encoded eight amino acids at the C-terminal end of the fusion protein. The positions of the forward and reverse primers (FP and RP) used to detect both TuYV-WT and TuYV-RT_GFP_ are indicated. The total length of each virus genome is reported. ▼: position of K437 (nt 4790), the last amino acid identified in the truncated readthrough (RT) protein; ▽: position of the *Nco*I and *Sal*I sites used in the cloning procedure; *: ORF5 stop codon.

In order to obtain a fluorescent TuYV virus, the sequence of the enhanced GFP [32] was introduced into the ORF5, encoding the RTD by replacing the C-terminal part of the RTD in the full-length TuYV infectious clone [33]. The RTD domain is involved in virus movement, although not strictly mandatory, but is essential for aphid transmission [17,22,25]. The resulting virus was named TuYV-RT_GFP_. The EGFP sequence was positioned 51 amino acids downstream of the lysine 437, the last amino acid of the RT* identified by mass spectrometry [41] (Figure 1). The required sequences for the readthrough mechanism of the CP stop codon [22] were maintained in the engineered virus. Due to the cloning strategy, TuYV-RT_GFP_ retained plasmid derived-sequences that introduced non-viral amino acids at the flanking extremities of the EGFP (five and two extra amino acids at the N- and C-terminus of the EGFP, respectively) (Figure 1). In addition, the last eight amino acids of the RT protein were conserved downstream of the C-terminal extremity of the EGFP.

**Figure 2:**
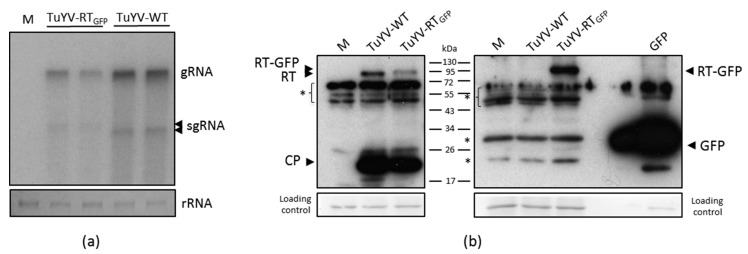
Viral replication and protein synthesis by TuYV-RT_GFP_ in *Chenopodium quinoa* protoplasts. (**a**) Northern-blot analysis of total RNA extracted from 120,000 protoplasts inoculated with DNA constructs corresponding to TuYV-RT_GFP_ or TuYV-WT, 48 h after inoculation. (M): mock-inoculated protoplasts. The position of the genomic (gRNA) and subgenomic (sgRNA) RNAs is indicated on the right. The loading control corresponds to methylene blue stained rRNAs; (**b**) Western blot analysis on total protein extracts prepared from TuYV-WT, TuYV-RT_GFP_, or mock-inoculated (M) protoplasts. As a control, proteins from *Nicotiana benthamiana* were infiltrated with agrobacteria containing a binary plasmid with the enhanced green fluorescent protein sequence (GFP). Two acrylamide gels were loaded in parallel with similar protoplast protein extracts and further transferred onto PVDF membranes. One membrane was incubated with antibodies raised against the TuYV coat protein (CP) revealing both CP and the readthrough protein (RT) (left panel) and the other was incubated with an antiserum directed against the GFP (right panel). *: non-specific products; Positions of the molecular markers (in kDa) are indicated. Ponceau red stained proteins serve as the loading control.

The infectivity of TuYV-RT_GFP_ was first addressed following the inoculation of *Chenopodium quinoa* protoplasts with the DNA construct containing the TuYV-RT_GFP_ genome under the control of the Cauliflower mosaic virus 35S promoter. Forty-eight hours after inoculation, both genomic and subgenomic RNAs of TuYV-RT_GFP_ were detected by Northern-blot (Figure 2a). Since the TuYV-RT_GFP_ genome was 215 nt longer than the wild-type genome (TuYV-WT), both RNA species migrated more slowly than the wild-type genomic and subgenomic RNAs. A reduction of TuYV-RT_GFP_ genomic and subgenomic RNAs accumulation ranging from 40 to 60% was observed compared with the TuYV-WT RNAs, suggesting that the introduction of the EGFP sequence into the TuYV genome slightly impaired virus replication in *C. quinoa* protoplasts (Figure 2a and data not shown). The detection of CP and RT-derived products was performed by western blot on the same *C. quinoa* protoplasts. Antibodies directed against the CP allowed The detection of the CP of 22 kDa and the RT protein fused to EGFP (RT:GFP) or the RT proteins in protoplasts infected either with TuYV-RT_GFP_ or TuYV-WT (Figure 2b, left panel). Indeed, both RT:GFP and RT proteins contain the CP at their N-terminus. CP and RT:GFP accumulations in the extract of protoplasts infected with TuYV-RT_GFP_ were slightly reduced compared to the wild-type virus, confirming the lower accumulation of the viral RNAs observed by Northern-blot (Figure 2a). The RT:GFP protein was also detected in the same extracts by antibodies directed against the GFP, confirming the hybrid nature of the protein (Figure 2b, right panel). Due to its larger size (76 kDa theoretical molecular weight), the RT:GFP migrated more slowly than the wild-type RT (74 kDa theoretical molecular weight), while both proteins displayed higher apparent molecular weights (around 97 and 95 kDa respectively) as already reported before for the RT protein [17,22]. Due to the presence of a non-specific signal around 30 kDa when using the anti-GFP serum, which could mask a potential release of the EGFP from the RT:GFP, the cleavage of the fusion protein in protoplasts could not be addressed with confidence. However, the equal intensity of the signal at 30 kDa in all protoplast extracts, including the one corresponding to TuYV-RT_GFP_, strongly suggests that free EGFP is not efficiently released from the RT:GFP (Figure 2b, right panel).

To further analyze the infectivity of TuYV-RT_GFP_ in whole leaves, the former TuYV-RT_GFP_ construct was introduced into a binary vector and inoculated into several plant species via *Agrobacterium tumefaciens*. The virus accumulation was evaluated by double antibody sandwich-ELISA and, as shown in Table 1, TuYV-RT_GFP_ accumulated in the agroinfiltrated leaves of *Nicotiana benthamiana*, *Montia perfoliata*, and *Arabidopsis thaliana* at a level close to TuYV-WT. Immunodetection performed on whole protein extracts showed the presence of both CP and RT:GFP proteins in the agroinfiltrated leaves of *N. benthamiana* (Figure S1). No cleavage product of the RT:GFP was observed.

In both assays (protoplasts and plants), western blot and/or DAS-ELISA analysis allowed the detection of the CP and RT proteins expressed from the sub-genomic RNA, confirming genuine virus replication since direct translation from the 35S promoter-derived transcripts could not result in the synthesis of both proteins. Taken together, these results showed that TuYV-RT_GFP_ was replication–competent in several host species and produced the expected RT:GFP fusion protein.

### 3.2. Viral Particle Formation

The formation of TuYV particles is a pre-requisite for virus systemic movement [42]. In order to address the ability of TuYV-RT_GFP_ to produce virus particles, virions were purified from *M. perfoliata* leaves infiltrated with either TuYV-RT_GFP_ or TuYV-WT. Typical isometric particles of ca. 25 nm of diameter were observed in the TuYV-RT_GFP_ purified preparation (Figure S2a). Western blot analysis showed that TuYV-RT_GFP_ and TuYV-WT particles contained comparable amounts of the truncated RT protein (RT*), suggesting that the EGFP inserted in the RT:GFP protein did not affect the processing of the fusion protein nor the incorporation of the RT* into virus particles (Figure S2b). When the blot was incubated with an antiserum directed against GFP, no signal was detected in the TuYV-RT_GFP_ purified viral extract, showing that the complete RT:GFP protein was not present in virions (data not shown). Together these results show that the protein compositions of TuYV-WT and TuYV-RT_GFP_ are similar. The RNA genome was extracted from both types of particles and amplified by reverse transcription-polymerase chain reaction (RT-PCR) using primers hybridizing within the TuYV 3’ noncoding sequence and within a central sequence of the RTD (primers FP and RP, Figure 1). The presence of a unique RT-PCR fragment of the expected size in the TuYV-RT_GFP_ virus extract (1085 bp) indicates that the viral genome has conserved the entire EGFP sequence while a DNA fragment of 870 bp is produced for TuYV-WT (Figure S2c).

### 3.3. Systemic Movement of TuYV-RT_GFP_

The capacity of TuYV-RT_GFP_ to traffic to non-inoculated leaves was addressed by agroinoculating young plants of three different species with the TuYV-RT_GFP_ binary construct. The presence of the virus in the upper leaves was assessed by DAS-ELISA two weeks later. TuYV-RT_GFP_ was detected in systemic leaves of *N. benthamiana*, *M. perfoliata*, and *A. thaliana*, but the percentage of infected plants differed depending on the plant species. The ability of TuYV-RT_GFP_ to reach systemic leaves in *N. benthamiana* was slightly reduced compared to TuYV-WT (50 to 76% of infected plants for TuYV-RT_GFP_ and 86 to 100% for TuYV-WT) while the infection rate of TuYV-RT_GFP_ in *M. perfoliata* was closed to the wild-type virus (mean of 71% of infected plants for TuYV-RT_GFP_ and 77.5% for TuYV-WT) (Table 2). In contrast, the TuYV-RT_GFP_ infection rate was lower in *A. thaliana* (22 to 38% of infected plants for TuYV-RT_GFP_ and 67 to 100% for TuYV-WT) (Table 2). TuYV-RT_GFP_ accumulation in the three plant species was always lower when compared to the wild-type virus accumulation, but, due to important variability in the DAS-ELISA values between plants, a statistical analysis could not be performed. In contrast to TuYV-WT, which induces a moderate leaf chlorosis on *N. benthamiana* or reddening symptoms on old leaves of *M. perfoliata*, no symptoms were observed on plants infected with TuYV-RT_GFP_. These results reinforce the hypothesis that the C-terminal part of the RT protein of TuYV is involved in symptom development [22]. *A. thaliana*, on the other hand, remained symptomless when infected with TuYV-WT or TuYV-RT_GFP_. Overall, these results showed that the replacement of the C-terminal part of the RT sequence by the EGFP sequence in the TuYV-RT_GFP_ does not hinder the systemic invasion of non-inoculated leaves.

**Figure 3:**
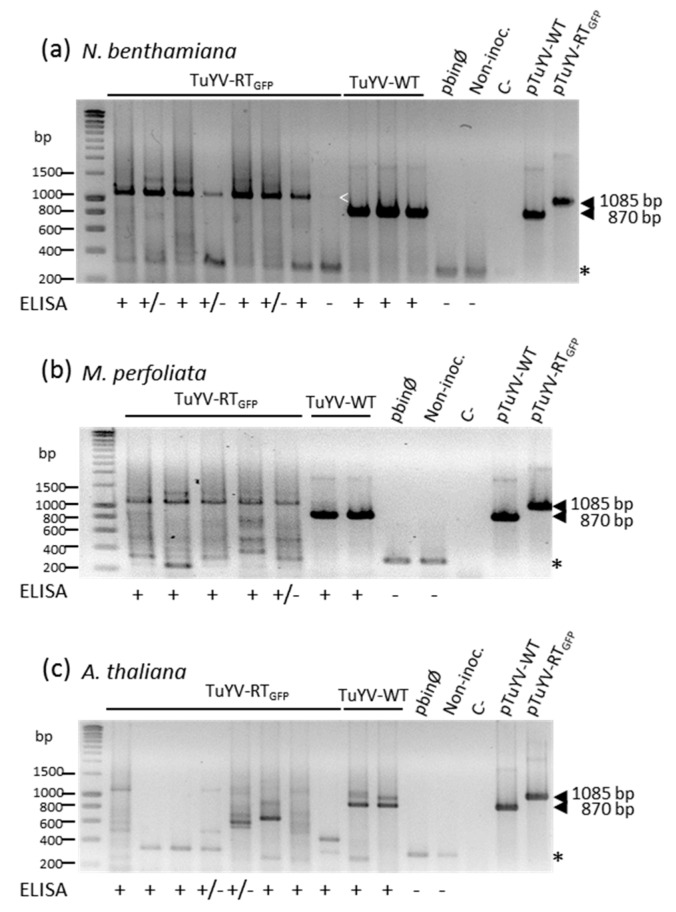
Analysis of viral progeny by reverse transcription-polymerase chain reaction (RT-PCR) in non-inoculated leaves of (**a**) *N. benthamiana*; (**b**) *M. perfoliata*; and (**c**) *A. thaliana* inoculated with TuYV-RT_GFP_ or TuYV-WT. Total RNA was isolated two weeks post-inoculation from plants which showed an ELISA OD value far above the threshold (ELISA+), just above the threshold (+/−), or below the threshold (−). A primer set was used to amplify a cDNA fragment corresponding to the 3’ end of TuYV-RT_GFP_ and TuYV-WT (FP and RP, Figure 1). PCR products were analyzed by gel electrophoresis and viewed after ethidium bromide staining. The positions of DNA markers are shown on the left. The size of expected fragments is also indicated. pbin-Ø: inoculation with a binary plasmid with no insert; Non-inoc.: non-inoculated plants. pTuYV-WT and pTuYV-RT_GFP_ refer to plasmids containing the viral sequences and serve as positive controls. C-: PCR control without cDNA. *: non-specific amplification; White open arrow-head: faint DNA amplification.

The stability of the inserted EGFP sequence in the viral genome was further addressed by analyzing the viral progeny in the three plant species by RT-PCR using the primers already described in the above paragraph. Total RNA was extracted from the systemic leaves of plants inoculated with TuYV-RT_GFP_ or TuYV-WT and considered to be infected according to the DAS-ELISA assay. Some plants for which infection with TuYV-RT_GFP_ was uncertain (DAS-ELISA value just above the threshold) were also included in the test. A major band of the expected size (1085 bp) was detected in the *N. benthamiana* plants undoubtedly infected with TuYV-RT_GFP_ (Figure 3a, ELISA OD+) and also in some plants where the presence of the virus was uncertain (Figure 3a, ELISA OD+/−). Surprisingly a very faint band was observed in the ELISA-negative plant (optical density (OD) value below the threshold) (white arrow head in Figure 3a). Since no band was present in the non-inoculated plant or the plant inoculated with the empty vector (Figure 3a), this may be explained by the lack of sensitivity of the immunodetection assay. These results show the conservation of the EGFP complete sequence in the viral progeny (Figure 3a). Additional minor bands of lower size and intensity were, however, present and may correspond to DNA fragments amplified from partially deleted genomes (Figure 3a). A similar situation was found in *M. perfoliata*, where the expected DNA fragment of 1085 bp was detected in all four plants that reacted positively to DAS-ELISA, but also in the plant for which infection was uncertain (Figure 3b, ELISA OD+/−). In addition, several by-products of lower size were also observed (Figure 3b). In *A. thaliana,* partial deletions or even the deletion of the entirety of the EGFP sequence in the viral genome were more frequent, suggesting important TuYV-RT_GFP_ genome rearrangements in this plant species. Nevertheless, the 1085 bp fragment corresponding to the complete TuYV-RT_GFP_ genome was detected in three out of eight plants assayed, indicating that the recombinant virus is able to systemically infect Arabidopsis (Figure 3c). The data presented in Figure 3 correspond to the representative experiments.

In a further experiment, the viral progeny of *N. benthamiana* (five plants) infected with TuYV-RT_GFP_ was analyzed by sequencing the DNA fragments produced after RT-PCR from total RNA using the aforementioned primers. The sequencing results confirmed the presence of the viral genome containing the entire EGFP sequence and some deleted forms in *N. benthamiana*.

In conclusion, these results show that TuYV-RT_GFP_ is able to spread in its full-size genome to non-inoculated leaves in the plant species tested. However, the stability of the recombinant genome varies between the plant species, with *N. benthamiana* being the host in which TuYV-RT_GFP_ undergoes the least rearrangements.

### 3.4. Aphid Transmissibility of TuYV-RT_GFP_

The ability of TuYV-RT_GFP_ to be transmitted by aphids was assessed by feeding *M. persicae* on an artificial medium containing similar amounts of TuYV-RT_GFP_ or TuYV-WT purified particles. The viruliferous aphids were then transferred to *N. benthamiana*, *M. perfoliata*, or *A. thaliana* for virus inoculation, and the test plants were analyzed by DAS-ELISA two weeks later. TuYV-RT_GFP_ was readily transmitted by aphids to all test plant species (Table 3). The integrity of the TuYV-RT_GFP_ genome present in the aphid-inoculated plants was tested as aforementioned by RT-PCR. As previously observed, the TuYV-RT_GFP_ genome containing the full-size EGFP sequence was detected together with genome-deleted forms (Figure S3).

### 3.5. In Vivo Tracking of TuYV-RT_GFP_ Following Agroinoculation or Aphid Transmission

**Figure 4:**
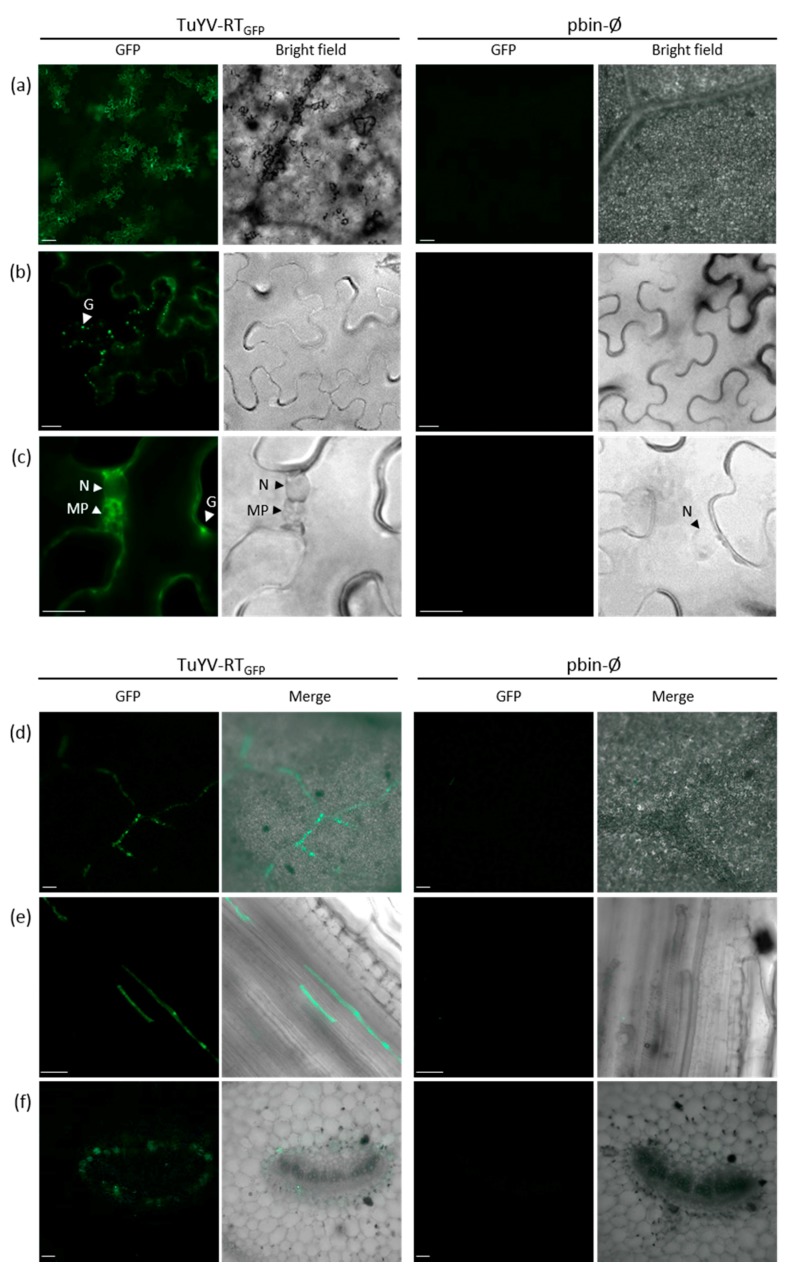
Cellular localization of EGFP fluorescence (**a**–**c**) in agroinoculated leaves and (**d**–**f**) in non-inoculated leaves of *N. benthamiana* agroinfiltrated with TuYV-RT_GFP_. *N. benthamiana* leaf (**d**) petiole, (**e**) longitudinal, or (**f**) transversal sections. The observations were performed with an epifluorescence microscope and in similar conditions on plant material infiltrated with pbin-Ø (last two columns on the right). Bright field observations are also shown to better locate the nucleus (second and fourth column for (**a**–**c**)), and the merge images of the bright field and the GFP emission are shown for (**d**–**f**) (second and fourth column). The scale bars are 20 µm for (**b**,**c**) and 100 µm for (**a**,**d**–**f**). (**f**) is an optical section obtained with structured illumination (ApoTome, Zeiss, Marly-le-Roi, France). N: nucleus; G: granules; MP: membrane proliferation.

To evaluate whether TuYV-RT_GFP_ could be used to track virus localization in infected plants, epifluorescence microscopy observations were conducted in the agroinoculated leaves of *N. benthamiana*. In most cases, groups of epidermal fluorescent cells were observed (Figure 4a). The fluorescence was present diffusely throughout the cytoplasm and in granules lining the plasma membrane (Figure 4b). Similar distribution has been reported for the GFP-labeled *Potato leafroll virus* (PLRV) in the inoculated leaves of *N. clevelandii* or *N. benthamiana* [30]. The microscopic observations also clearly revealed fluorescent foci in perinuclear localizations that could be membrane-derived (Figure 4c). The observations were then carried out on non-inoculated leaves of infected *N. benthamiana*, *M. perfoliata*, and *A. thaliana* two to three weeks after agroinoculation. The GFP fluorescence was detected along the vasculature of the plant as observed in a leaf from TuYV-RT_GFP_-infected *N. benthamiana* at a low magnification (Figure 4d).

A similar pattern of fluorescence labeling the plant vasculature was also obtained when observing whole plants using an epifluorescence macroscope (Figure S4a,b). At a higher magnification with an epifluorescence microscope, observations of the longitudinal sections of petioles or of isolated vein networks showed fluorescence in elongated cells in the veins of the three plant species (Figure 4e, Figure S4c,d) or in phloem cells surrounding the xylem (Figure 4f). Importantly, no fluorescence was visible in the non-phloem cells of TuYV-RT_GFP_ infected plants (Figure 4d–f). No signal was monitored in similar sections or leaf discs from *N. benthamiana*, *M. perfoliata*, or *A. thaliana* infiltrated with the empty binary vector (Figure 4a–f and Figure S4c,d, right panels).

**Figure 5:**
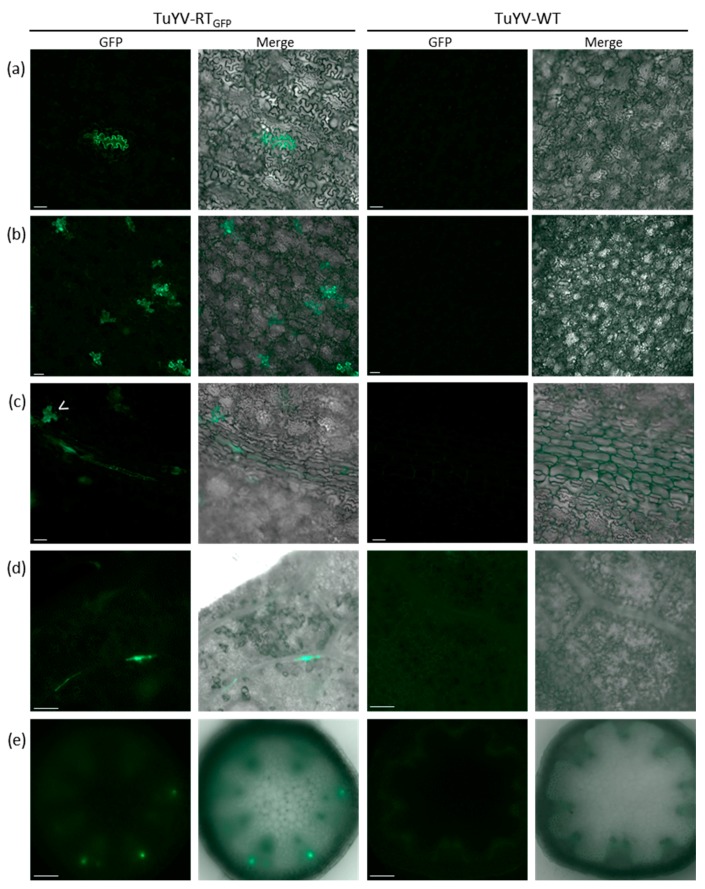
Cellular localization of EGFP fluorescence in the inoculated or systemic leaves of infected plants after aphid delivery of TuYV-RT_GFP_ or TuYV-WT. (**a**–**c**) Cellular localization of EGFP in *M. perfoliata* inoculated leaves five days after aphid inoculation. Cellular localization of EGFP in a non-inoculated (**d**) leaf and (**e**) stem of *A. thaliana* two weeks after virus delivery by aphids. The observations were performed with an epifluorescence microscope and in similar conditions on plant material inoculated with TuYV-WT (last two columns on the right). Merge images of the bright field and the GFP emission are also shown (second and fourth column). White open arrow-head: isolated infected epidermal cells. Scale bars are 100 µm.

When TuYV-RT_GFP_ was delivered to *M. perfoliata* by aphids, isolated fluorescent epidermal cells with a notched shape were observed in the confined zone where the aphids were deposited (Figure 5a–c). These cells likely represent cells punctured by *M. persicae* during the probing phase, in which the virus was delivered and replicated. Similarly, as was reported for a GFP-tagged PLRV genome [30], the fluorescence was always confined to isolated cells and never extended to adjacent cells, thus confirming the inability of TuYV to move to neighbouring non-phloem cells. Similar observations of isolated fluorescent cells were made on the aphid-inoculated leaves of *A. thaliana* (not shown). Interestingly, on the aphid-inoculated leaves of *M. perfoliata*, in addition to the isolated epidermal cells (open arrow in Figure 5c), elongated consecutive fluorescent cells lining the veins were also observed in a plane underneath, suggesting viral replication in phloem cells (Figure 5c). These infected phloem cells likely represent the true primary infection sites from which TuYV may spread to adjacent cells.

We then addressed the ability of TuYV-RT_GFP_ to invade non-inoculated leaves following aphid inoculation. To facilitate fluorescence observation in the veins, the epidermis of *A. thaliana* leaves was peeled off. Fluorescent cells were detected along the veins (Figure 5d), and fluorescent foci were also observed in a transversal section of a flowering stem of infected *A. thaliana* (Figure 5e). In this case, the fluorescent foci were located at the periphery of the stem section where the vascular bundles are located (Figure 5e). No signal was monitored in the leaves of plants inoculated with aphids carrying TuYV-WT (Figure 5a–e, right panels).

Free GFP was reported to be able to spread by passive diffusion through expanding tissues [43,44]. In order to confirm that the fluorescence observed in TuYV-RT_GFP_-infected leaves was indeed associated to virus replication, the leaf samples were first observed by epifluorescence microscopy and then tested by DAS-ELISA or RT-PCR to detect the presence of the virus or the viral RNA. Leaf discs were collected from 15 non-inoculated leaves of three *N. benthamiana* infected with TuYV-RT_GFP_ (positive by DAS-ELISA two weeks after agroinoculation). In 12 out of 15 leaves analyzed, the detection of the virus correlated perfectly with the fluorescence observation. In two leaves however, although no fluorescence was observed, the full-size viral genome containing the EGFP sequence was detected in one of the samples by RT-PCR, likely denoting a lack of sensitivity of the fluorescence detection system. Finally, one leaf displayed several fluorescent foci, but no viral genome was detected (data not shown).

## 4. Discussion

Tracking fluorescent viruses in plants has proven to be a valuable tool to study the critical steps of the viral cycle involved in virus replication or movement in compatible or incompatible host interactions [45,46,47,48,49,50,51]. This approach was also developed to observe the initial stages of virus infection following aphid transmission of a non-persistently transmitted virus [52]. In addition, cellular and subcellular localizations of GFP-fused viral proteins were conducted after inserting the GFP sequence into a viral genome [53,54,55,56]. However, the insertion of the fluorochrome sequence into a viral genome has always been a challenge since it can reduce the infectivity of the fluorescent virus or generate virus-deleted forms impaired in their ability to move systemically [57,58,59,60,61]. Introducing non-viral sequences into a viral genome can become particularly difficult for viruses with icosaedric particles, as they are often subject to high constraints of genome size for efficient encapsidation [62,63].

The present study reports the first fluorescently tagged virus in the *Luteoviridae* family that successfully develops a systemic infection in several plant species after agroinoculation or aphid transmission. In the engineered TuYV-RT_GFP_, the GFP is expressed as a fusion protein with the N-terminal part of the RT protein. Importantly the full-size recombinant genome was recovered in the viral progeny from *N. benthamiana* and *M. perfoliata*, indicating its stability in these host plants.

Attempts to obtain a GFP-tagged infectious clone of Potato leafroll virus (PLRV), a member of the *Luteoviridae* family, were formerly reported. A clone in which the 48 last amino acids of the RT protein were removed and replaced by the GFP sequence was shown to be infectious, but the genome proved to be unstable as large deletions encompassing the GFP insert were reported [30]. Although the strategy developed by Nurkiyanova et al. shares similarities with the one we used to generate TuYV-RT_GFP_ [30], the engineered PLRV-GFP had a larger genome size compared to TuYV-RT_GFP_, which could explain some genome instability and therefore its inability to develop a fluorescent systemic infection in plants after aphid transmission or agroinoculation. An engineered Beet mild yellowing virus (BMYV, family *Luteoviridae*) tagged with GFP was also obtained, but its infectivity was only evaluated in *A. thaliana* protoplasts after the inoculation of viral-derived transcripts [31].

The TuYV-RT_GFP_ engineered virus was able to develop a systemic infection in three plant species tested, *N. benthamiana*, *M. perfoliata*, and *A. thaliana*, when delivered by agrobacteria or by aphids. The presence of a full-length RT protein, although not mandatory, is required for efficient polerovirus systemic movement [19,24,25]. The lack of the C-terminal part of the RT protein may therefore be responsible for the lower infectivity of TuYV-RT_GFP_ observed more specifically in *A. thaliana*. Alternatively, the GFP moiety present at the C-terminal might generate some physical hindrance and/or epitope hindrance during viral movement. Moreover, we cannot exclude that the additional non-viral and non-EGFP sequences in the TuYV-RT_GFP_ construct can have an effect on virus replication and systemic movement.

Full-length GFP-tagged viral genomes were easily recovered in the systemic leaves of infected *M. perfoliata* and *N. benthamiana* plants. However, deleted viral genomes, the abundance of which varied depending on the plant species, were also retrieved. In particular, viral genomic rearrangements, resulting in the loss of the GFP sequence integrity, were more frequent in *A. thaliana*, but the presence of these deleted viral genomes did not prevent the invasion of the plant by the complete TuYV-RT_GFP_ genome. Whether these genome alterations are related to host-specific functions of the RT protein requires further investigation. Aphid transmission of TuYV-RT_GFP_ was also successfully achieved as fluorescent foci were detected in systemic leaves. This confirms the dispensable nature of the C-terminal domain of the polerovirus RTD for aphid transmission [22,24].

In our study, TuYV-RT_GFP_ was able to produce *in planta* the fusion protein RT:GFP and form virions with a protein composition similar to the wild-type virus. As expected, the EGFP moiety was absent from virions, since only the cleaved form RT*, which does not contain the GFP, is present in viral particles. Surprisingly, Nurkiyanova et al. [30] reported the presence of the RT:GFP fusion protein in particles of PLRV, which suggests that, in this particular case, the insertion of the GFP sequence into the PLRV genome might have impaired the correct cleavage of the RT protein.

Microscopic observations performed on agroinoculated *N. benthamiana* leaves localized the RT:GFP produced by TuYV-RT_GFP_ diffusely in the cytoplasm or as granules at the periphery of the cell. Fluorescent structures were also observed in the vicinity of the nucleus. These structures looked like membrane proliferations and could be sites of viral replication or encapsidation [64]. Considering that the RT:GFP is a C-terminal truncated form of the complete RT and resembles the RT* incorporated into virions, this represents the first sub-cellular observations of the truncated RT protein expressed from a viral clone. Similar localizations were obtained for the almost complete RT:GFP from PLRV. Although this protein localizes to the nucleolus when expressed alone, it was excluded from the nucleus in the viral context [65]. Microscopic observations performed on *M. perfoliata* leaves inoculated with TuYV-RT_GFP_ viruliferous aphids identified fluorescent cells corresponding to sites of aphid probing in the epidermis and in infected elongated phloem cells. While the epidermal cells were always isolated, which is in accordance with the inability of the virus to move from these cells to adjacent cells, the fluorescent phloem cells were observed to be aligned in a row, suggesting viral trafficking between vascular cells. In a few instances, the fluorescent epidermal and phloem cells were observed in close proximity, suggesting that they might have got infected from the same aphid, and could therefore represent the presumed primary infection sites in the vasculature. When searching for food, the aphid punctures the epidermal cells for probing before inserting intercellularly its stylets to reach the phloem cells, where the virus is inoculated and replicates. It has been shown that several cells along the stylet path can be punctured [66], but, in our experiments, we did not observe fluorescent cells other than epidermal and phloem cells.

In newly developed leaves of *N. benthamiana*, *M. perfoliata*, and *A. thaliana* plants following inoculation by agrobacteria or aphids, the fluorescently tagged TuYV was also strictly observed in the cells of the vasculature. As the entire RT:GFP protein was not found incorporated into TuYV-RT_GFP_ particles, the observed fluorescence likely corresponds to viral multiplication sites. It could be conceivable that some of the fluorescent signals also arise from free GFP, released after the cleavage of the RT:GFP to produce the RT* incorporated into virions, spreading by passive diffusion through expanding tissues [43,44]. However, this hypothesis seems unlikely since we showed that in almost all non-inoculated leaves, fluorescence emission correlated with virus detection. In addition, in contrast to previous observations on the post-phloem transport of the GFP [43,44], fluorescence trafficking from the phloem cells into adjacent mesophyll cells was never detected in our study. This observation indicates that the GFP fragment cleaved from RT:GFP is likely degraded, similar to the C-terminal part of the RT that has never been detected by western blot in infected tissues. This reinforces our assumption that the GFP fluorescence observed in TuYV-RT_GFP_ infected plants is indeed the consequence of virus replication rather than the passive translocation of free GFP. The detection of full-size TuYV-RT_GFP_ genomes by RT-PCR in systemic tissue is an additional proof of the long-distance movement of TuYV-RT_GFP_.

The recombinant TuYV-RT_GFP_ described in this study is a powerful tool that will contribute to future advances in the functional analysis of viral and host genes involved in the polerovirus cycle in planta and in polerovirus transmission by aphids.

## Figures and Tables

**Table 1:** Detection by double antibody sandwich-ELISA of TuYV-RT_GFP_ in inoculated leaves of several plant species.

	*Nicotiana benthamiana*		*Montia perfoliata*		*Arabidopsis thaliana*	
	Nb pl. inf./pl. inoc. ^1^	OD ± SD ^2^	Nb pl. inf./pl. inoc. ^1^	OD ± SD ^2^	Nb pl. inf./pl. inoc. ^1^	OD ± SD ^2^
**TuYV-RT_GFP_**	4/4	2.59 ± 0.42	3/3	2.11 ± 0.15	4/4	1.80 ± 0.87
**TuYV-WT**	3/3	2.87 ± 0.05	4/4	2.61 ± 0.20	4/4	1.83 ± 0.59
**pbin-Ø**	0/2	0.16 ± 0.00	0/2	0.14 ± 0.01	0/2	0.11 ± 0.01
**Non-inoc. ^3^**	0/2	0.15 ± 0.01	0/2	0.14 ± 0.00	0/1	0.10 ± 0.00

^1^ Number of plants infected/plants inoculated. A plant is considered infected when the optical density (OD) value of the leaf extract is above the mean OD values of three non-infected plants and three times the standard deviation of these values. ^2^ The mean absorbance value at 405 nm of the agroinfiltrated leaves four to seven days after inoculation ± Standard Deviation. ^3^ Non-inoculated plants. pbin-Ø: empty binary vector.

**Table 2:** Detection by double antibody sandwich (DAS)-ELISA of TuYV-RT_GFP_ in the non-inoculated leaves of three plant species.

	*Nicotiana benthamiana*						*Montia perfoliata*				*Arabidopsis thaliana*			
	*Exp.1*		*Exp.2*		*Exp.3*		*Exp.1*		*Exp.2*		*Exp.1*		*Exp.2*	
	Nb pl. inf./pl. inoc. ^1^	OD ± SD ^2^	Nb pl. inf./pl. inoc. ^1^	OD ± SD ^2^	Nb pl. inf./pl. inoc. ^1^	OD ± SD ^2^	Nb pl. inf./pl. inoc. ^1^	OD ± SD ^2^	Nb pl. inf./pl. inoc. ^1^	OD ± SD ^2^	Nb pl. inf./pl. inoc. ^1^	OD ± SD ^2^	Nb pl. inf./pl. inoc. ^1^	OD ± SD ^2^
**TuYV-RT_GFP_**	4/8 (50%)	1.12 ± 0.59	13/17 (76%)	0.52 ± 0.23	4/7 (57%)	0.54 ± 0.12	4/6 (67%)	1.42 ± 0.69	6/8 (75%)	0.70 ± 0.34	6/16 (38%)	0.31 ± 0.13	2/9 (22%)	0.69 ± 0.16
**TuYV-WT**	8/8 (100%)	1.86 ± 0.30	4/4 (100%)	0.63 ± 0.22	6/7 (86%)	1.61 ± 0.79	4/6 (67%)	1.58 ± 0.62	7/8 (88%)	1.90 ± 0.24	8/8 (100%)	1.70 ± 0.16	4/6 (67%)	1.40 ± 1.23
**pbin-Ø**	0/2	0.14 ± 0.30	0/1	0.11	0/1	0.15	0/2	0.13 ± 0.00	0/2	0.11 ± 0.01	0/2	0.10 ± 0.00	0/2	0.12 ± 0.00
**Non-inoc.^3^**	0/3	0.14 ± 0.00	0/3	0.13 ± 0.01	0/3	0.16 ± 0.01	0/2	0.11 ± 0.00	0/3	0.10 ± 0.01	0/3	0.10 ± 0.01	0/3	0.11 ± 0.01

^1^ Number of plants infected/plants inoculated. ^2^ Mean absorbance values at 405 nm of the non-inoculated leaves of the infected plants two weeks after inoculation ± Standard Deviation. A plant is considered infected when the optical density (OD) value of the leaf extract is above the mean OD values of three non-infected plants plus three times the standard deviation of these values. ^3^ Non-inoculated plants. The OD value corresponds to the absorbance of the non-inoculated plant.

**Table 3:** TuYV-RT_GFP_ transmission by *Myzus persicae* from purified virus.

	*N. benthamiana ^1^*	*M. perfoliata* ^1^		*A. thaliana* ^1^
	*Exp.1*	*Exp.1*	*Exp.2*	*Exp.1*
	Nb pl.inf./pl. inoc. ^2^	Nb pl.inf./pl. inoc. ^2^	Nb pl.inf./pl. inoc. ^2^	Nb pl.inf./pl. inoc. ^2^
**TuYV-RT_GFP_**	10/10	9/10	5/10	4/10
**TuYV-WT**	9/10	10/10	10/10	10/10
**Non-inoc. ^3^**	0/3	0/3	0/3	0/3

^1^ *Nicotiana benthamiana*, *Montia perfoliata*, or *Arabidopsis thaliana* were used as plant tests in the transmission experiments. ^2^ Number of plants infected/plants inoculated. Double antibody sandwich (DAS)-ELISA was performed on non-aphid inoculated leaves two weeks after inoculation. A plant is considered infected when the optical density (OD) value of the leaf extract is above the mean OD values of three non-infected plants plus three times the standard deviation of these values. ^3^ Non-inoculated plants.

## Supplementary Materials

The following are available online at www.mdpi.com/1999-4915/9/5/166/s1, Figure S1: Analysis of TuYV-RT_GFP_ multiplication in infiltrated leaves of *N. benthamiana*, Figure S2: Analysis of virus particles purified from *M. perfoliata* leaves infiltrated with TuYV-RT_GFP_ or TuYV-WT, Figure S3: Analysis of viral progeny by RT-PCR in non-inoculated leaves of *M. perfoliata* and *A. thaliana* inoculated by aphids with TuYV-RT_GFP_ or TuYV-WT, Figure S4: Cellular localization of EGFP fluorescence emitted by TuYV-RT_GFP_ inoculated to plants by agroinoculation, Table S1: List of primers.
