# Supplementary material for: Systemic Propagation of a Fluorescent Infectious Clone of a Polerovirus Following Inoculation by Agrobacteria and Aphids
Source: Viruses. 2017 Jun 29;9(7):166. doi: 10.3390/v9070166 (PMC5537658; doi:10.3390/v9070166)
Supplement: Supplementary file 1 [file viruses-09-00166-s001.zip › Table S1 TuYV-GFP.pdf]

Table S1: List of primers

Primer name	Pimer sequence 5'> 3'
Reverse primer RP	ATTGTCGACACCGAAGTGCCGTA
Forward primer FP	AGGCTCATCCGGTTCCGT
5’RT-Nco.fw	GGTCTAGACCATGGCAGTCTCAACC
5’RT-Bam.rev	GGGGATCCTCTTTTGCAGCGTGTCAG
3’RT-Eco.fw	GAGAATTCGGCGAAGACCCCGAC
3’RT-Sal.rev	CAGGTCGACTAGACACCGAAGTG
EGFP-Bam.fw	GTGGATCCTTATGGTGAGCAAGGGC
EGFP-Eco.rev	TCGAATTCCTTGTACAGCTCGTCC
