# Supplementary material for: Systemic Propagation of a Fluorescent Infectious Clone of a Polerovirus Following Inoculation by Agrobacteria and Aphids
Source: Viruses. 2017 Jun 29;9(7):166. doi: 10.3390/v9070166 (PMC5537658; doi:10.3390/v9070166)
Supplement: Supplementary file 1 [file viruses-09-00166-s001.zip › TuYV-GFP FigS1.pdf]

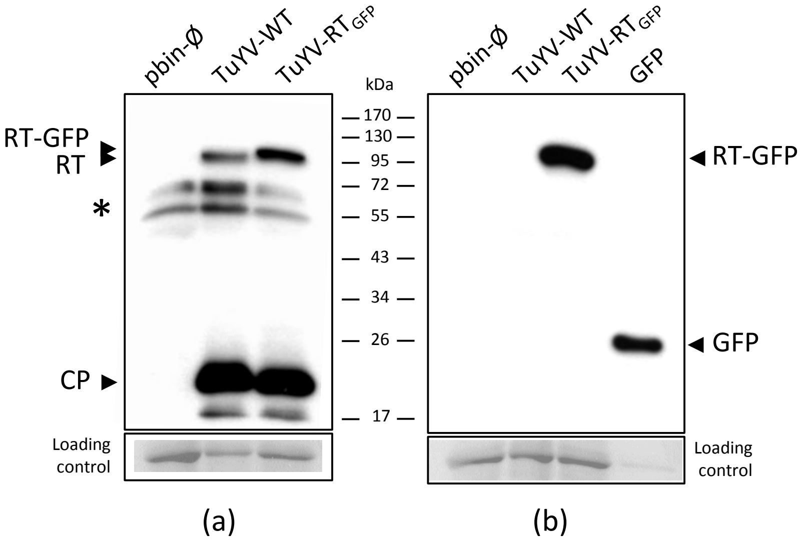


**Figure S1:** Analysis of TuYV-RT$_{GFP}$ multiplication in infiltrated leaves of *N. benthamiana*. Western blot analysis of total protein extracts prepared 5 days post-inoculation with agrobacteria containing TuYV-RT$_{GFP}$ or TuYV-WT constructs or a binary plasmid with no insert (pbin-Ø). Two acrylamide gels were loaded in parallel with the same protein extracts and transferred onto membranes. Membrane (**a**) was incubated with antibodies raised against TuYV-CP and the membrane (**b**) was incubated with an antiserum directed against GFP. *: non-specific products. Position of the molecular markers (in kDa) is indicated. Ponceau red stained proteins serve as a loading control.
