# Supplementary material for: Systemic Propagation of a Fluorescent Infectious Clone of a Polerovirus Following Inoculation by Agrobacteria and Aphids
Source: Viruses. 2017 Jun 29;9(7):166. doi: 10.3390/v9070166 (PMC5537658; doi:10.3390/v9070166)
Supplement: Supplementary file 1 [file viruses-09-00166-s001.zip › TuYV-GFP FigS2.pdf]

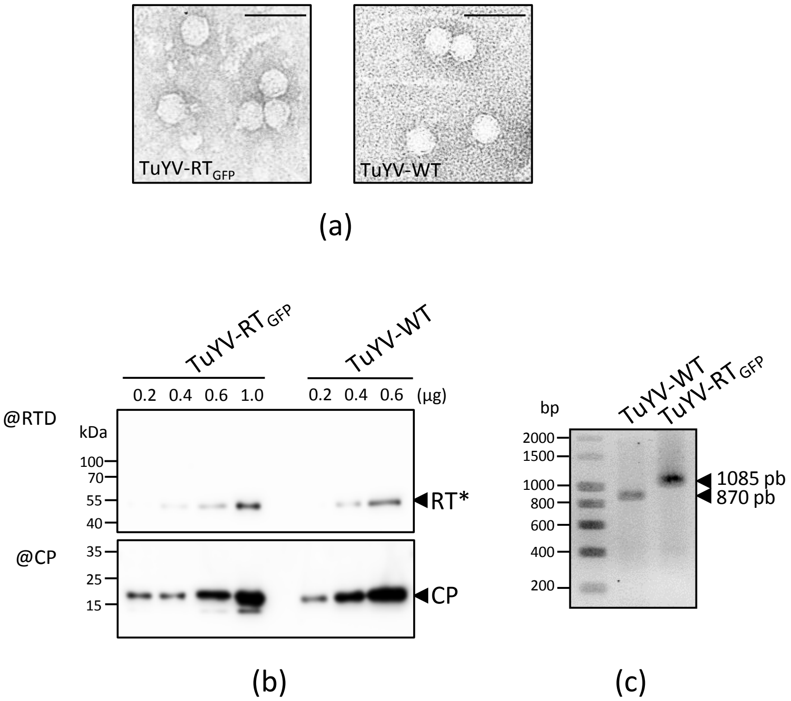

Figure S2: Analysis of virus particles purified from *M. perfoliata* leaves infiltrated with TuYV-RT_{GFP} or TuYV-WT. (**a**) Virus particles were observed by ISEM using, for coating, antibodies raised against TuYV particles. Scale bars are 50 nm; (**b**) The protein composition of TuYV-RT_{GFP} and TuYV-WT particles was analyzed by western blot. The upper part of the blot was probed with antibodies directed against TuYV-RTD and the lower part with a TuYV-CP-specific antiserum. Increasing amounts of virus (from 0.2 to 0.6 or 1.0 µg) were loaded for each virus. Position of the molecular markers (in kDa) is indicated on the left; (**c**) RT-PCR analysis of the viral RNA encapsidated into virions of TuYV-RT_{GFP} and TuYV-WT using FP and RP primers (Fig. 1). The PCR fragments were analysed by gel electrophoresis and viewed after ethidium bromide staining. Position of the molecular markers (in bp) is indicated on the left. Sizes of the expected fragments are also indicated.
