# Supplementary material for: Systemic Propagation of a Fluorescent Infectious Clone of a Polerovirus Following Inoculation by Agrobacteria and Aphids
Source: Viruses. 2017 Jun 29;9(7):166. doi: 10.3390/v9070166 (PMC5537658; doi:10.3390/v9070166)
Supplement: Supplementary file 1 [file viruses-09-00166-s001.zip › TuYV-GFP Figure S3 revised.pdf]

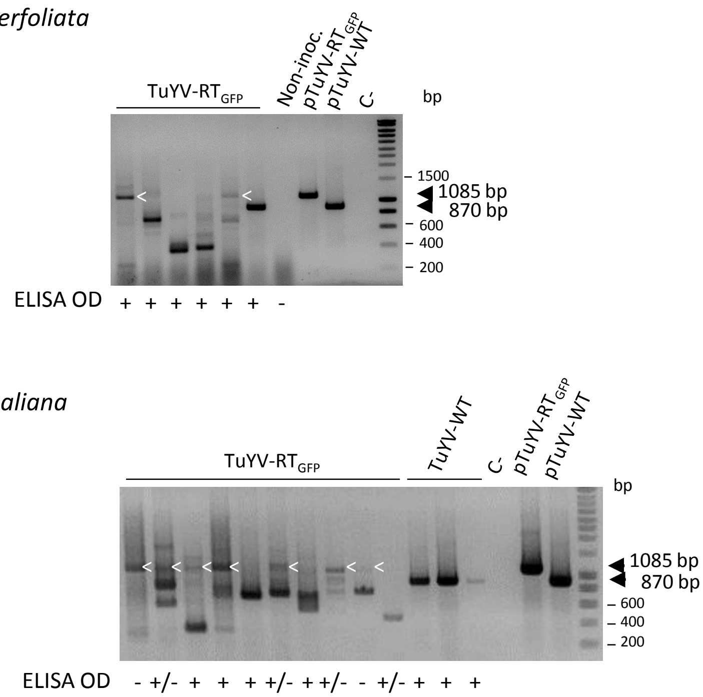

Figure S3: Analysis of viral progeny by RT-PCR in non-inoculated leaves of (**a**) *M. perfoliata* and (**b**) *A. thaliana* inoculated by aphids with TuYV-RT_{GFP} or TuYV-WT. Total RNA was isolated two weeks post-inoculation from plants which showed an ELISA OD value far above the threshold (ELISA +), just above the threshold (+/-) or below the threshold (-). A primer set was used to amplify a cDNA fragment corresponding to the 3′ end of TuYV-RT_{GFP} and TuYV-WT (FP and RP, Fig. 1). PCR products were analyzed by gel electrophoresis and viewed after ethidium bromide staining. For *M. perfoliata*, the results shown in (a) correspond to the Exp.1 in Table 3 in which 6 out of the 9 plants positive on ELISA and inoculated with TuYV-RT_{GFP} have been analyzed by RT-PCR. For *A. thaliana*, results shown in (b) correspond to the Exp. 1 in Table 3 in which all the plants inoculated with TuYV-RT_{GFP} have been analyzed. Position of DNA markers is shown on the right. The size of expected fragments is also indicated. Non-inoc.: non-inoculated plants. pTuYV-WT and pTuYV-RT_{GFP} are referring to plasmids containing the viral sequences and serve as positive controls. C-: PCR control without cDNA. *: non-specific amplification; White open arrow-head: expected DNA amplification.
