# Supplementary material for: Systemic Propagation of a Fluorescent Infectious Clone of a Polerovirus Following Inoculation by Agrobacteria and Aphids
Source: Viruses. 2017 Jun 29;9(7):166. doi: 10.3390/v9070166 (PMC5537658; doi:10.3390/v9070166)
Supplement: Supplementary file 1 [file viruses-09-00166-s001.zip › TuYV-GFP FigS4 revised.pdf]

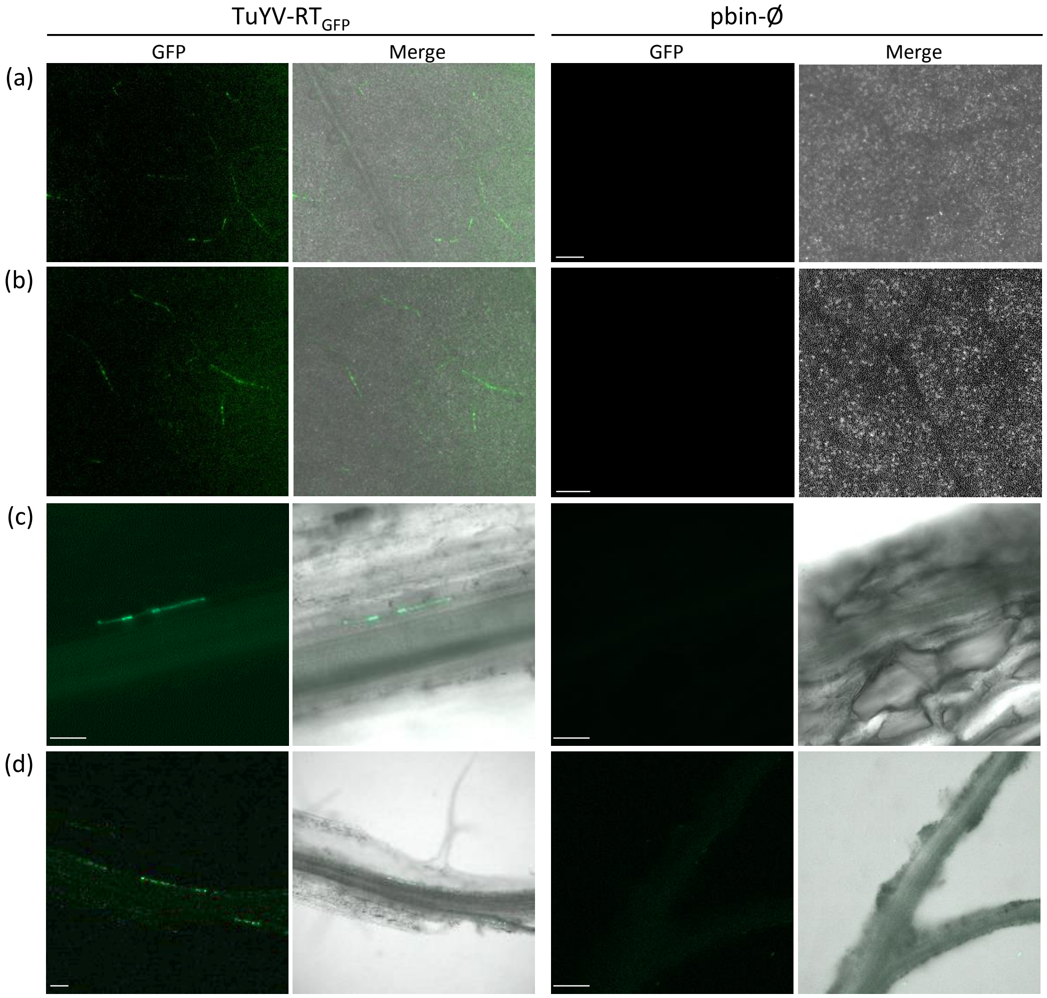

Figure S4: Cellular localization of EGFP fluorescence emitted by TuYV-RT$_{GFP}$ inoculated to plants by agroinoculation. (**a**)-(**b**) Non-inoculated leaves of *N. benthamiana* were observed with an epifluorescence macroscope directly on whole plants without destructive sampling. Observations were performed in similar conditions on plant material infiltrated with pbin-Ø (last two columns on the right). Merge images of the bright field and GFP emission are also shown (second and fourth column). (**c**) Longitudinal section of a petiole from non-inoculated leaves of *M. perfoliata* infected with TuYV-RT$_{GFP}$; (**d**) veins extracted from non-inoculated leaves of *A. thaliana* infected with TuYV-RT$_{GFP}$. Observations in (**c**)-(**d**) were done using an epifluorescence microscope and correspond to optical sections obtained with structured illumination (ApoTome, Zeiss). Observations were made in similar conditions on plant material infiltrated with pbin-Ø (last two columns on the right). The merge images of the bright field and GFP emission are shown (second and fourth column). Scale bars are 500 µm for (**a**)-(**b**) and 100 µm for (**c**)-(**d**).
